# Severe food allergy in children. Omalizumab as an alternative treatment to elimination diet

**DOI:** 10.1186/2045-7022-1-S1-P55

**Published:** 2011-08-12

**Authors:** María Pena Peloche, Miguel Hinojosa Macías, Soledad Terrados Cepeda, Pilar Berges Gimeno, Gema Vanesa Sánchez Moreno, Emilio Álvarez-Cuesta

**Affiliations:** 1 Ramón y Cajal, Allergy Division, Madrid, Spain

## Background

Severe food allergy is a life threatening condition with few therapeutics alternatives. A pilot study with omalizumab, as an alternative treatment in persistent milk and egg allergy, has been conducted.

## Methods

Children above 6 years with IgE-mediated allergy to milk or egg. Anaphylaxis history and positive skin prick test (SPT) and sIgE levels against food. Informed consent was required.

Patients received a sixteenth week treatment with anti IgE Omazilumab. SPT and single blind food challenges (SBFC) were performed after the treatment. Adverse reactions were registered.

## Results

Ten patients were included, 3 patients suffered allergy to milk and 7 to egg . They had a positive SBFC or a previous history of food transgressions with reactions in the last six months. All patients received anti-IgE treatment without any significant adverse reaction. After 16 weeks, eight patients underwent to SBFC and complete tolerance was confirmed in four patients. Four patients presented a positive SBFC. All subjects reached a higher dose threshold than baseline SBFC. Two patients rejected SBFC. 6 patients with anaphylactic allergy were allocated in a specific oral tolerance induction (SOTI) with anti IgE treatment. During SOTI, only one subject experienced some mild side-effects, 2 patients successfully completed the treatment, and 4 patients still continue with their SOTI procedure without any significant reactions during in-patient administered doses or out-patient maintenance doses.

## Conclusions

Anti-IgE therapy has proven an effective and safe measure in the treatment of the persistent and severe allergy to milk and egg, whether used as monotherapy or as an adjuvant measure to the process of desensitization to food, making it safer and faster procedure.

